# Why We Move: Social Mobility Behaviors of Non-Disabled and Disabled Children across Childcare Contexts

**DOI:** 10.3389/fpubh.2016.00204

**Published:** 2016-09-21

**Authors:** Samuel W. Logan, Samantha Mae Ross, Melynda A. Schreiber, Heather A. Feldner, Michele A. Lobo, Michele A. Catena, Megan MacDonald, James C. Galloway

**Affiliations:** ^1^Social Mobility Laboratory, College of Public Health and Human Sciences, Oregon State University, Corvallis, OR, USA; ^2^Ergonomics and Safety Laboratory, Department of Mechanical Engineering, University of Utah, Salt Lake City, UT, USA; ^3^Department of Physical Therapy, University of Illinois at Chicago, Chicago, IL, USA; ^4^Move to Learn Innovation Laboratory, Department of Physical Therapy, University of Delaware, Newark, DE, USA; ^5^Children and Youth with Disabilities Laboratory, College of Public Health and Human Sciences, Oregon State University, Corvallis, OR, USA; ^6^Go Baby Go Program, Department of Physical Therapy, University of Delaware, Newark, DE, USA

**Keywords:** locomotion, disability, early intervention, physical activity, play behaviors

## Abstract

**Background:**

Social mobility is defined as the co-occurrence of self-directed locomotion and direct peer interaction. Social mobility is a product of dynamic child–environment interactions and thus likely to vary across contexts (e.g., classroom, gymnasium, and playground).

**Purpose:**

The purpose of this present study was to examine differences in children’s social mobility: (1) across contexts by age and (2) between non-disabled and disabled children.

**Method:**

Participants (*n* = 55 non-disabled and three disabled children; M_age_ = 3.1 years, SD = 1.4) were video recorded within a university-based early learning center. Children were recorded for 20 min in each context: classroom, gymnasium, and playground. A 15-s momentary time sampling method was used to code social mobility, the simultaneous occurrence of self-directed locomotion, and direct peer interaction. This variable was calculated as percent time within each context.

**Results:**

A planned Friedman’s rank ANOVA (*n* = 55), stratified by age, indicated that older children (3–5 years old) differed across contexts in their social mobility [χ^2^(2) ~ 7.3–10.5, *p* < 0.025], whereas younger children (1–2 years old) were similar across contexts. Social mobility was significantly lower in the classroom compared with the playground and gymnasium (with no difference between the latter contexts) for older children. Visual analysis confirmed that disabled children (*n* = 3) engaged in substantially less time in social mobility (average 0–1%), compared with non-disabled, age-similar peers (2–3 years old average 1–12%) across all contexts.

**Conclusion:**

A substantial gap exists between non-disabled and disabled children for social mobility. There is an increase in magnitude and variability of social mobility around age three that suggests the gap between non-disabled and disabled children will continue to widen.

## Introduction

Development in childhood is dynamic, non-linear, and embedded within day-to-day experiences (1). The dynamic systems perspective of child development illustrates this complexity as an interaction between constraints at the individual level (e.g., body function and structure, motivation), interpersonal level (e.g., caregiver and peer social relationships, attachments), and environmental level (e.g., accessibility for exploration and engagement) (1). Another related concept that can be applied to child development is grounded cognition, which places an emphasis on an individual’s engagement in perceptual-motor experiences and their formative role in children’s developmental trajectory across cognitive, social, and communication domains (2). Both dynamic systems and grounded cognition illustrate how the intersection of developmental domains within children’s daily life shapes and defines their health and well-being. Physical activity is one type of perceptual-motor experience that has been linked to social interactions (3).

Physical activity engagement is a dynamic and interactive experience for children (4, 5). Physical activity is defined as “…any bodily movement produced by skeletal muscles resulting in energy expenditure” [(4, 6), p. 126]. However, an alternative and multidimensional definition of physical activity that captures the social component of movement has emerged. It describes physical activity as the “individual agency of activity related to movement, in relation to energy expenditure and social engagement” (i.e., voluntary, self-directed, and purposeful exploration and play) (7, 8). A substantial gap exists between non-disabled and disabled children in frequency, duration, and intensity of physical activity (3, 9). Limited research has examined how physical activity, social interactions, and play are interrelated during early childhood (10, 11).

Emergent research has examined the relationship between physical activity and social interactions in toddlers (10). Longitudinal data have shown spikes in the development of social interactions with mothers (e.g., vocalization and gesturing) and object use following achievement of motor milestones such as crawling and walking (12–14). Delays in gross motor development are analogously predictive of less mature forms of social play and language in later childhood (11, 15, 16). This research has lent to a consensus that motor skills emerge prior to, and are positively related to the future development of social and communication skills (10, 16, 17). A critical aspect that remains unknown is the co-occurrence of these behaviors in terms of developmental trajectories using time-locked observations. This knowledge would further our understanding of how these domains intersect and influence development at the moment-to-moment level that is emphasized by dynamic systems theory and grounded cognition.

This project is an extension of the original work published by Logan et al. (3). Logan et al. (3) explored the time-locked co-occurrence of physical activity and social interactions in 2-year-old non-disabled children (*n* = 23), alongside disabled children (*n* = 2), while engaged in routine experiences within an early learning center. Physical activity was broadly defined to include trunk and limb movements and/or locomotion (i.e., moving at least three feet in any direction). Social interactions were defined to include parallel play (i.e., children within three feet of each other but not directly interacting), direct peer interaction, and direct adult interaction. Results suggest that the two disabled children engaged in less frequent and less variable physical activity and social interactions, and these behaviors were less likely to co-occur in comparison to non-disabled children. This research provides initial insight into the dynamic nature of physical activity and play behaviors and highlights potential disparities between non-disabled and disabled children that we should aim to minimize *via* intervention, assistive technology, and community design.

The current study is a follow-up to the original work of Logan et al. (3) and includes 55 non-disabled children age 1–5 years old and three disabled children. The data of 21 2-year-old non-disabled children and 2 disabled children (Child A and B) from Logan et al. (3) are included in the current study. The current study extends Logan et al. (3) in three ways. First, the current study focuses on a specific behavior termed “social mobility,” defined as children’s simultaneous engagement in self-directed locomotion and direct peer interaction. In the original work, the occurrence and co-occurrence of physical activity, play, and object-use behaviors were reported but social mobility behaviors were not reported. Second, the current study examines social mobility behaviors separately across three contexts of the childcare setting (classroom, gymnasium, and playground). In the original work, physical activity, play, and object-use behaviors were combined across childcare settings and were not reported separately. Third, a wider age range of non-disabled children is included (1–5 years old). In the original work, only 21 non-disabled 2-year-old children were included. The specific aim of the present study is to examine the differences in children’s social mobility (1) across contexts by age and (2) between non-disabled and disabled children. It is hypothesized that the occurrence of social mobility will vary across contexts and be greater among older children. Further, it is hypothesized that disabled children will engage in social mobility less often than non-disabled children across all contexts.

## Materials and Methods

### Participants

Participants included 55 non-disabled children aged 1–5 years old (M = 3.1 years, SD = 1.4 years; 29 females). There were a similar number of children within each age group: 1-year old (*n* = 10), 2-year olds (*n* = 11), 3-year olds (*n* = 9), 4-year olds (*n* = 13), and 5-year olds (*n* = 12). Participants’ parents reported their ethnicities as: Caucasian (47%), African-American (39%), Asian (12%), and Middle Eastern (2%).

Participants also included three disabled children. They will be referred to as “Child A,” “Child B,” and “Child C” to protect their identities. Cognitive function was not measured for any participants, thus we cannot rule out a cognitive influence on each child’s behaviors observed for the present study. Child A was a Caucasian girl (age = 31 months old). Her primary diagnosis was cerebral palsy (Gross Motor Function Classification System – level IV) with secondary diagnoses of microcephaly, hypotonia, and cortical vision impairment/persistent fetal vascular syndrome. She had the ability to interact with cause-and-effect toys such as those that light up or make sounds. She could also distinguish between different types of animals and colors. Furthermore, she was able to recognize and respond to different people such as her teacher, physical therapist, and parent/caregivers. She had the ability to roll on the floor and to sit on the floor with close supervision and hands-on support but was unable to pull to stand or walk (even with use of assistive technology). She used a manual wheelchair throughout the day for her seating and positing needs but required adult assistance for propulsion. She vocalized often, but did not say words. Child A received services related to language, fine and gross motor, and cognitive skills.

Child B was an African-American boy (age = 33 months old). His primary diagnosis was developmental delay with secondary diagnoses of mild hearing loss in the left ear and epilepsy (type: electrical status epilepticus during sleep). Similar to Child A, he responded to cause-and-effect toys and had the ability to categorize objects and responded to the people with whom he interacted with on a regular basis. He also had the ability to respond to and follow instructions, although there were demonstrated behavioral issues related to self-regulation. He was able to independently sit, stand, and walk without assistance. However, his movements were ataxic, and he usually required physical and/or verbal prompts and assistance to initiate movements. He vocalized often but said few words – generally names of people or objects. Child B received services related to language and gross motor skills.

Child C was an Asian boy (age = 4.1 years old). His medical history includes diagnoses of ventricular septal defect, bilateral clubfeet, and bilateral peroneal neuropathy. At 44 months of age, he underwent surgery to treat a tethered spinal cord. He wore solid bilateral ankle foot orthoses and used forearm crutches for mobility. He walked without assistance using forearm crutches over a variety of surfaces – hallway, grass, mulch, inclines, and declines. He received physical therapy services twice weekly, and his family received consultation once per month per his individualized education plan. The focus of physical therapy was to improve trunk, upper extremity, and lower extremity strength within the context of promoting function and participation within life situations. Child C did not receive services related to language, fine motor, or cognitive skills.

### Procedure

The study procedure for the current study is reported in detail elsewhere (3). Approval from the university Institutional Review Board and written parent/guardian consent was obtained prior to data collection. In brief overview, children were video recorded while attending a university-based early learning center. Each child was video recorded for 20 min while engaged in routine activities in the classroom, gymnasium, and playground (i.e., 60 min total). The three disabled children were recorded for an extended time of 60 min per context. Physical activity and social interactions were assessed *via* video analysis and direct observation measures.

Observational behavioral coding was conducted by an experienced coding team, with an 85% intra- and inter-rater agreement established for 10% of recordings *a priori* using the ratio of [agreements/(agreements + disagreements) × 100] to establish a percentage of agreement. A 15-s momentary time sampling method was used to code the occurrence of locomotion, peer interaction, and social mobility. This method includes a 5-s observation period and 10-s record period that results in four observations per minute. Each non-disabled child had approximately 240 total behavioral observations evenly divided between the classroom, gymnasium, and playground. Each disabled child had approximately 720 total behavioral observations evenly divided between the classroom, gymnasium, and playground.

### Behavioral Assessment

#### Assessment of Locomotion

The observed system for recording physical activity in children-preschool version (18) was used to assess children’s physical activity intensity level. Intensity categories included stationary/motionless, stationary with trunk and limb movement, slow-easy, moderate, and fast movement. Locomotion was defined as three steps, or the equivalent, in any direction using any modality (walking, crawling, or running), at any intensity level (slow, moderate, or fast).

#### Assessment of Social Interaction

The Howes Peer Play Scale (19) was used to assess play behaviors including solitary play, parallel play, peer interaction, and teacher interaction (verbal or physical). Parallel play occurred when a child is within close proximity (<3 feet) to a peer or teacher but is not directly interacting. Peer interaction includes direct verbal and/or physical interaction that is initiated by or directed toward the key child by a peer.

#### Assessment of Social Mobility

Social mobility was defined as the simultaneous co-occurrence of self-directed locomotion and direct peer interaction, and operationalized as percent of assessment time observed.

### Planned Data Analysis

It was expected that data would violate the underlying assumptions of normality and homogeneity given developmental variability within this age group, the small sample size, and our ordinal outcome variable (percent time spent in social mobility). Thus, a planned non-parametric statistical approach is described below and was conducted using SPSS (version 22, 2013). Statistical methods are not presently available to compare individuals to the group; therefore, visual analysis was used to compare non-disabled and disabled children. This approach offers valuable insight into real-world experiences of observed behaviors between non-disabled and disabled children in their natural settings (3).

Our planned analysis to examine social mobility across contexts and age groups (aim 1) was threefold. First, Spearman’s correlations (rho) were calculated to examine the association between age in years and social mobility, independent of context. Significant correlations between age in years and social mobility supported subsequent analyses conducted with data stratified by age. Second, a Friedman’s analysis of variance by ranks test was conducted to examine group differences in social mobility rankings across contexts and within stratified age groups. *Post hoc* comparisons were calculated (Pairwise Wilcoxon sum rank tests). Third, a Spearman’s correlation (rho) was calculated to examine the association between individual social mobility rankings across contexts relative to age-similar peers. The strength of the correlation coefficients were interpreted based on Cohen’s *d* guidelines (small = *r* > 0.10; moderate = *r* > 0.30; and strong = *r* > 0.50) (20). Visual analysis of the three disabled children allowed for comparison to non-disabled children across contexts and age groups (aim 2).

## Results

The Shapiro–Wilks test indicated that the data significantly deviated from a normal distribution for all contexts (classroom: *W* = 0.80, *p* < 0.001; gymnasium: *W* = 0.80, *p* < 0.001; playground: *W* = 0.82, *p* < 0.0001). The normality assumption was also violated when tested within specific age sub-groups. The Levene’s statistic indicated that the variance of the data for each context was significantly heterogeneous (classroom: *W* = 0.80, *p* < 0.001; gymnasium: *W* = 0.80, *p* < 0.001; playground: *W* = 0.82, *p* < 0.001). As anticipated, a non-parametric approach with stratified age groups and exclusion of disabled children from group analysis was required to address our specific aims.

**Figure 1:**
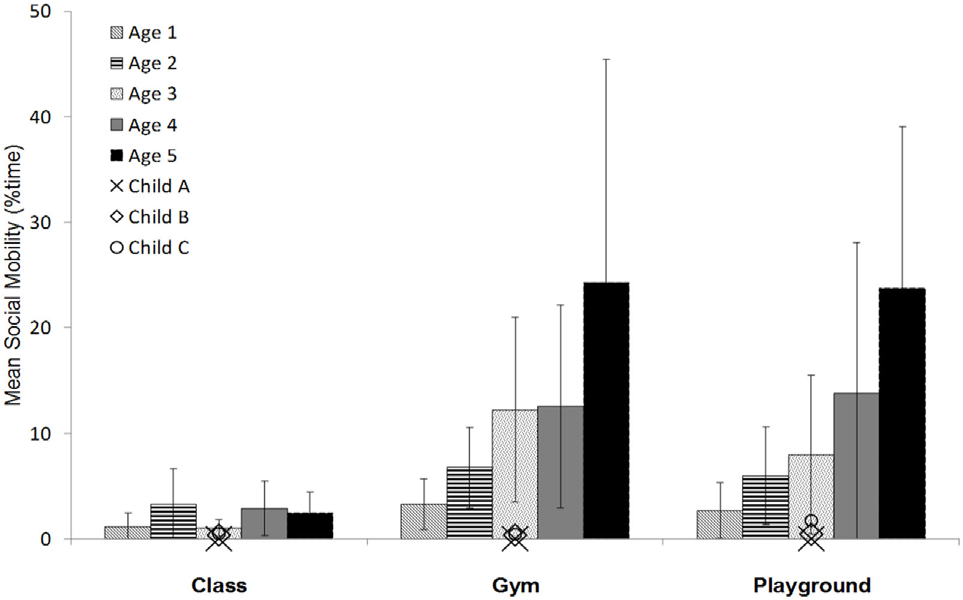
**Comparison of mean social mobility (% time) across 1- to 5-year-old age groups and contexts (classroom, gymnasium, and playground) and in relation to individual disabled children (Child A, B, and C)**.

On average, children spent the greatest percentage of time in social mobility within the gymnasium (M = 11.8%; SD = 13.2%), compared to the playground (M = 10.8%; SD = 12.3%) and the classroom (M = 2.2%; SD = 2.3%). Similar trends were observed when stratified by age (Table 1; Figure 1). This indicates that the average time spent engaged in social mobility varies by context, regardless of children’s age.

**Table 1:** **Social mobility (% time) group means, SD, SE, mean rank, and results of Friedman’s Rank Test contexts by age**.

Age	Context	*N*	Mean			Mean rank
			Mean	SD	SE	
1	Classroom	10	1.1	0.8	0.3	1.5
	Gymnasium	10	3.3	2.4	0.8	2.3
	Playground	10	2.7	2.6	0.8	2.2
						χ^2^(2) = 3.80, ω = 0.19, *p* = 0.15
2	Classroom	11	3.3	3.3	1.0	1.4
	Gymnasium	11	6.7	3.9	1.2	2.3
	Playground	11	6.0	4.5	1.4	2.3
						χ^2^(2) = 5.90, ω = 0.27, *p* = 0.05
3	Classroom	9	1.0	0.8	0.3	1.3
	Gymnasium	9	12.2	9.0	3.0	2.4
	Playground	9	8.0	7.4	2.5	2.3
						χ^2^(2) = 7.37, ω = 0.41, *p* = 0.03*
4	Classroom	13	2.8	2.7	0.7	1.38
	Gymnasium	13	12.9	9.6	2.7	2.23
	Playground	13	13.0	11.8	3.4	2.38
						χ^2^(2) = 7.84, ω = 0.30 *p* = 0.02*
5	Classroom	12	2.5	1.9	0.6	1.25
	Gymnasium	12	24.3	20.3	5.9	2.25
	Playground	12	23.7	15.7	4.5	2.50
						χ^2^(2) = 10.50, ω = 0.44, *p* < 0.01*
Total	Classroom	55	2.2	2.3	0.3	1.4
	Gymnasium	55	12.3	13.2	1.8	2.3
	Playground	55	11.2	12.3	1.7	2.3
						χ^2^(2) = 34.065, ω = 0.310, *p* < 0.001*

*Not inclusive of disabled children, mean rank based on % time of individual children within given age group (Friedman’s Rank Sum Test)*.

*Significant at an a priori alpha = 0.05.

A significant rank order Spearman’s rho correlation between age and social mobility was observed within the gymnasium [*r*_s_ (54) = 0.48, *p* < 0.001] and the playground [*r*_s_ (54) = 0.55, *p* < 0.001]. A non-significant Spearman’s rho correlation was observed within the classroom [*r*_s_ (54) = 0.22, *p* > 0.05]. This indicates that significant, moderate to strong relationships exist between age and social mobility in the gymnasium and the playground. Therefore, subsequent analyses were conducted using age-stratified groups.

A Friedman’s analysis of variance by ranks test revealed a significant difference in rankings of children’s social mobility across contexts for age groups 3, 4 and 5 years [3-year olds: X^2^ (2) = 7.37, ω = 0.41, *p* < 0.05; 4-year olds: X^2^ (2) = 7.84, ω = 0.30, *p* < 0.05; 5-year olds: X^2^ (2) = 10.50, ω = 0.44, *p* < 0.01] (Figure 1). Mean rankings of 1- and 2-year olds did not differ across contexts (Figure 1). This indicates that older children (3- to 5-year olds) differ across context in the percentage of time spent engaged in social mobility, whereas younger children (1- and 2-year olds) tend to be similar in social mobility regardless of context.

*Post hoc* Pairwise Wilcoxon Sum Rank tests were conducted to examine specific differences in social mobility between contexts for older age groups 3- to 5-year olds. Our analysis revealed that social mobility in the classroom was significantly lower than in both the gymnasium and on the playground for all older children (see Table 2). Social mobility did not significantly differ between the playground and the gymnasium for any of the older age groups (see Table 2). This indicates that, among older children, social mobility engagement is similar within the gymnasium and playground, with the percent of time in social mobility in both these contexts being significantly greater than in the classroom.

**Table 2:** **Wilcoxon pairwise comparisons**.

Age	Gymnasium vs. classroom	Playground vs. classroom	Playground vs. gymnasium
3	*Z* = −2.497, *p* = 0.013*	*Z* = −2.547, *p* = 0.011*	*Z* = −0.866, *p* = 0.386
4	*Z* = −2.760, *p* = 0.006*	*Z* = −2.830, *p* = 0.005*	*Z* = 0.00, *p* = 1.00
5	*Z* = −2.824, *p* = 0.005*	*Z* = −2.903, *p* = 0.004*	*Z* = −0.078, *p* = 0.937

*Significant at an a priori alpha = 0.05.

The individual data points for social mobility of the three disabled children were plotted against the mean for each age group of typically developing peers (Figure 1). Visual analysis of this graph revealed a disparity in social mobility, regardless of context, relative to age-similar non-disabled children. However, the disabled children (aged 2–4 years) displayed trends in their variability of social mobility across contexts similar to younger peers. In other words, the individual disabled children did not differ in their time spent in social mobility between contexts, a trend consistent with 1- and 2-year-old non-disabled children (Figure 1). Of concern, increased social mobility in the gymnasium and on the playground was not observed for the 4-year old with a disability, as would be expected based on trends observed for age-similar peers. This indicates that disabled children may experience increasing gaps in social mobility compared to non-disabled children as they age.

## Discussion

The present study describes the differences in children’s social mobility: (1) across contexts by age and (2) between non-disabled and disabled children. Our analysis indicated that the average social mobility within each context significantly differed by age. Older children spent a greater percent of time engaged in social mobility compared to younger peers. These results are consistent with our primary hypothesis. Additionally, younger and older children differed in the variability of social mobility across contexts. Among younger children, the time spent in social mobility was comparable in the classroom, gymnasium, and playground, whereas older children spent significantly more time engaged in social mobility within the gymnasium and playground than in the classroom. In support of our second hypothesis, disabled children engaged in less social mobility compared to non-disabled children within all contexts, with evidence that this gap increases with age.

The variability of social mobility for older, but not younger children, across contexts is consistent with expected developmental trajectories in the motor and social domains. On average, the onset of self-directed locomotion occurs between 10 and 14 months of age for non-disabled children (21). Children continue to advance in several skills within the motor domain across early childhood. Advancements from basic to more advanced cognitive and language skills similarly emerge during early childhood (17, 22). Children simultaneously advance in the quality of their self-directed movement from one place to another, their use of this self-directed locomotion for more advanced social interactions, and in maturity of their language and social skills (23). This transition is also reflected in the shift from primarily engaging in parallel play – individual play in the presence of a peer without direct interaction – that is observed among young toddlers, to interactive peer play during preschool years (3). However, comparable social mobility in the classroom between 3- and 5-year olds suggests that developmental trajectories accounts only partially for this behavior.

The observed differences across contexts for older children align with the dynamic systems and grounded cognition frameworks. Children are expected to interact dynamically with, and be influenced by contextual and intra- and interpersonal factors. For the observed children, the physical space dimensions, tasks provided, and teacher expectations in the classroom likely contributed to the reduced social mobility. Alternatively, the gymnasium and the playground share environmental similarities in terms of physical space to run and play, the presence of developmentally appropriate equipment and structures, and the opportunity to engage in tasks involving greater physical activity and verbal interaction. There also tend to be more open play and encouragement for peer interaction within these activity settings. There is a need to identify the key aspects of the environment that facilitate and hinder physical activity and social interactions to further support the development of social mobility as children age. Our results indicate the need to also consider characteristics unique to each age group in future discussions on this topic.

At an individual level, children who demonstrate high levels of social mobility are likely to move and engage more across all contexts. Disabled children, however, may engage in these behaviors less than their non-disabled peers, regardless of context. There is also an evident shift in social mobility behaviors relative to age, with younger children demonstrating less social mobility than older children. Thus, the gap between non-disabled and disabled children may continue to widen in early childhood as the normative bar is raised, and as physical play environments incorporate more complex and potentially inaccessible activities.

### Limitations and Future Research

There are limitations of the current study. This study used a cross-sectional research design. This is an important first step in describing social mobility behaviors of children, yet only provides a snapshot in time regarding the individual and group differences of the social mobility behaviors of children. Future research may include the use of a longitudinal research design that will allow for the observation of the emergence and developmental trends of social mobility. Also, we did not formally assess cognition for the children in this study, so it is not clear if/how cognition impacted children’s social mobility. It is possible that children with differing cognitive abilities may engage differently in social mobility. Future research can address this question by including a larger sample size of children with a variety of cognitive and physical abilities to determine how these abilities may influence the children’s engagement in social mobility. Another limitation is the low sample size (*n* = 3) of disabled children. It is important to acknowledge the heterogeneity inherent in the wide range of disabilities of disabled children that were included in the current study. Results should be interpreted cautiously and without generalization to larger populations. It is difficult to conduct studies with a large sample size of disabled children in real-world settings such as early childcare centers. Further, fully inclusive practices are not well established in a majority of early childcare settings, therefore. curriculum design, teacher training and education, and environmental design may also be factors that influence social mobility for disabled children in particular (24–26). Future research may continue to examine the role of the environment, such as accessibility of play structures and toys, as well as the role of early childcare professionals to facilitate social mobility opportunities for disabled children.

## Conclusion

In the original work of Logan et al. (3), co-occurrences of specific physical activity types and levels were reported with play behaviors, including parallel play, peer and teacher interactions. The current work reports the specific and time-locked co-occurrence of children’s simultaneous engagement in self-directed locomotion and direct peer interaction. Results of the current study extend the findings of Logan et al. (3) by providing a better understanding of how locomotion specifically facilitates peer interaction, rather than play behaviors at a broad level. The findings from this study suggest that children’s individual social mobility differs by context. Specifically, a child’s social mobility level in the classroom is distinctly different from their engagement level within settings that are less guided by adults and that allow for increased movement, vocalization, and play, such as the gymnasium or playground. Further, disabled children display less social mobility behaviors, regardless of context, when compared to non-disabled children. The gap in participation between these groups is expected to increase with time. Future studies are needed to examine the impact of social mobility on future health and developmental outcomes, as well as to examine the environments and interactions, external to the child, that influence these behaviors. The long-term goal is to identify mechanisms that facilitate the development of motor and social skills among children, enhance movement and social interactions, improve inclusive practices and accessible environmental designs, and ultimately reduce the gap in participation between non-disabled and disabled children.

## Author Contributions

SL, JG, and MS contributed significantly to the conceptualization and development of this research. JG oversaw the project, with SL and MS serving as leads for collection and behavioral coding analysis. SR conducted statistical analysis and led the writing of this manuscript. All authors contributed significantly to the writing and development of this manuscript.

## Conflict of Interest Statement

The authors declare that the research was conducted in the absence of any commercial or financial relationships that could be construed as a potential conflict of interest. The reviewer NK and handling Editor declared their shared affiliation, and the handling Editor states that the process nevertheless met the standards of a fair and objective review.

## References

[1] Thelen E Dynamic systems theory and the complexity of change. Psychoanal Dialogues (2005) 15(2):255–83. 10.1080/10481881509348831

[2] Lobo MA Harbourne RT Dusing SC Mccoy SW. Grounding early intervention: physical therapy cannot just be about motor skills anymore. Phys Ther (2013) 93(1):94–103. 10.2522/ptj.20120158 23001524 PMC3538987

[3] Logan SW Shrieber M Lobo MA Pritchard B George L Galloway JC Real-wold performance: physical activity, play and object-related behaviors of toddlers with and without disabilities. Pediatr Phys Ther (2015) 27(4):433–41. 10.1097/PEP.0000000000000181 26397093

[4] World Health Organization. Physical Activity Fact Sheet [Internet]. World Health Organization (2016). Available from: http://www.who.int/mediacentre/factsheets/fs385/en/

[5] Kang L Palisano RJ King GA Chirarello LA. A multidimensional model of optimal participation of children with physical disabilities. Disabil Rehabil (2014) 36(20):1735–41. 10.3109/09638288.2013.863392 24325580

[6] Caspersen CJ Powell KE Christenson GM. Physical activity, exercise, and physical fitness: definitions and distinctions for health-related research. Public Health Rep (1985) 100(2):126–31. 3920711 PMC1424733

[7] Malina RM. Top 10 research questions related to growth and maturation of relevance to physical activity, performance, and fitness. Res Q Exerc Sport (2014) 85(2):157–73. 10.1080/02701367.2014.897592 25098012

[8] Ross SM Case L Leung W Aligning physical activity measures with the international classification of functioning, disability and health framework for childhood disability. Quest (2016) 1–15. 10.1080/00336297.2016.1145128

[9] Leung GPK Chan CCH Chung RCK Pang MYC. Determinants of activity and participation in preschoolers with developmental delay. Res Dev Disabil (2011) 32(1):289–96. 10.1016/j.ridd.2010.10.005 21036536

[10] Leonard HC Hill EL Review: the impact of motor development on typical and atypical social cognition and language: a systematic review. Child Adolesc Ment Health (2014) 19(3):163–70. 10.1111/camh.12055 32878369

[11] Kennedy-Behr A Rodger S Mickan S. A comparison of the play skills of preschool children with and without developmental coordination disorder. OTJR Occup Particip Health (2013) 33(4):198–208. 10.3928/15394492-20130912-03 24652028

[12] Clearfield MW Learning to walk changes infants’ social interactions. Infant Behav Dev (2011) 34(1):15–25. 10.1016/j.infbeh.2010.04.008 20478619

[13] Wang MV Lekhal R Aarø LE Schjølberg S. Co-occurring development of early childhood communication and motor skills: results from a population-based longitudinal study. Child Care Health Dev (2014) 40(1):77–84. 10.1111/cch.12003 22970997

[14] Walle EA Campos JJ. Infant language development is related to the acquisition of walking. Dev Psychol (2014) 50(2):336. 10.1037/a0033238 23750505

[15] Kennedy-Behr A Rodger S Mickan S Physical and social play of preschool children with and without coordination difficulties: preliminary findings. Br J Occup Ther (2011) 74(7):348–54. 10.4276/030802211X13099513661199

[16] Libertus K Violi DA. Sit to talk: relation between motor skills and language development in infancy. Front Psychol (2016) 7:475. 10.3389/fpsyg.2016.00475 27065934 PMC4815289

[17] Piek JP Dawson L Smith LM Gasson N. The role of early fine and gross motor development on later motor and cognitive ability. Hum Mov Sci (2008) 27(5):668–81. 10.1016/j.humov.2007.11.002 18242747

[18] Brown WH Pfeiffer KA McIver KL Dowda M Almeida JM Pate RR Assessing preschool children’s physical activity: the observational system for recording physical activity in children-preschool version. Res Q Exerc Sport (2006) 77(2):167–76. 10.1080/02701367.2006.10599351 16898273

[19] Howes C Matheson CC Sequences in the development of competent play with peers: social and social pretend play. Dev Psychol (1992) 28(5):961 10.1037/0012-1649.28.5.961

[20] Cohen J Statistical Power Analysis for the Behavioral Sciences. 2nd ed Hillsdale, New Jersey: Erlbaum (1988).

[21] Onis M. WHO motor development study: windows of achievement for six gross motor development milestones. Acta Paediatr (2006) 95(S450):86–95. 10.1080/08035320500495563 16817682

[22] Iverson JM Developing language in a developing body: the relationship between motor development and language development. J Child Lang (2010) 37(02):229–61. 10.1017/S0305000909990432 20096145 PMC2833284

[23] Campos JJ Anderson DI Barbu-Roth MA Hubbard EM Hernstein MJ Witherington D Travel broadens the mind. Infancy (2000) 1(2):149–219. 10.1207/S15327078IN0102_1 32680291

[24] Odom SL Preschool inclusion what we know and where we go from here. Top Early Child Spec Educ (2000) 20(1):20–7. 10.1177/027112140002000104

[25] Harper LV McCluskey KS Teacher–child and child–child interactions in inclusive preschool settings: do adults inhibit peer interactions? Early Child Res Q (2003) 18(2):163–84. 10.1016/S0885-2006(03)00025-5

[26] Rafferty Y Piscitelli V Boettcher C The impact of inclusion on language development and social competence among preschoolers with disabilities. Except Child (2003) 69(4):467–79. 10.1177/001440290306900405

